# The identification of novel small extracellular vesicle (sEV) production modulators using luciferase‐based sEV quantification method

**DOI:** 10.1002/jex2.62

**Published:** 2022-09-27

**Authors:** Aki Yamamoto, Yuki Takahashi, Shinsuke Inuki, Shumpei Nakagawa, Kodai Nakao, Hiroaki Ohno, Masao Doi, Yoshinobu Takakura

**Affiliations:** ^1^ Department of Biopharmaceutics and Drug Metabolism Graduate School of Pharmaceutical Sciences Kyoto University Sakyo‐ku Kyoto Japan; ^2^ Department of Bioorganic Medicinal Chemistry and Chemogenomics Graduate School of Pharmaceutical Sciences Kyoto University Sakyo‐ku Kyoto Japan; ^3^ Department of Systems Biology Graduate School of Pharmaceutical Sciences Kyoto University Sakyo‐ku Kyoto Japan

**Keywords:** autophagy, extracellular vesicles, luminescence, modulators, nanovesicles, quantification, screening

## Abstract

Small extracellular vesicles (sEVs) are nano‐sized vesicles secreted from various cells that contain bioactive metabolites and function as key regulators for intercellular communication. sEVs modulate diverse biological and pathological processes in the body, and the amount of circulating sEVs has been reported to correlate with certain disease progression. Therefore, the identification of small molecular compounds that can control sEV production may become a novel therapeutic strategy. In this study, a rapid, highly sensitive sEV quantification method utilizing fusion proteins consisting of Gaussia luciferase (gLuc) reporter protein and sEV markers (CD63 and CD82) was developed. A total of 480 compounds were screened to identify potent inducers and inhibitors of gLuc activity. Two novel compounds, KPYC08425 and KPYC12163, showed significant and dose‐dependent changes in gLuc activity with minimal cytotoxicity based on the LDH assay. The efficacy of these two compounds was further evaluated by protein quantification of the isolated sEVs. Further evaluation of KPYC12163 suggested that the autolysosomal pathway may be involved in its inhibitory effect on sEV production.

## 1 INTRODUCTION

Small extracellular vesicles (sEVs) are a heterogeneous group of lipid‐bilayered nanovesicles secreted by nearly all cell types that play a key role in intercellular communication (el Andaloussi et al., 2013; Lässer et al., 2011; Théry et al., 2018). They contain nucleic acids, proteins, lipids and other biologically active metabolites derived from the producing cells, which are delivered to neighbouring and distant cells to modulate diverse biological and pathological processes in the body (van Niel et al., 2018; Zaborowski et al., 2015). The nature and abundance of sEVs are dependent on the type of sEV‐producing cells, as well as its physiological or pathological state and ultimately influence its function in the body. Normally, sEVs function to maintain homeostasis and regulate immune responses (Tkach & Théry, 2016; Yáñez‐Mó et al., 2015); however, sEVs can also play a role in disease onset and progression. For example, in the case of cancer, tumour cell‐derived sEVs are known to facilitate disease progression by promoting tumorigenesis, immune escape and metastasis (Becker et al., 2016).

Owing to its role in disease progression, the identification of small molecular compounds that can regulate sEV production is becoming increasingly popular as a novel therapeutic strategy (Catalano & O'Driscoll, 2020; Datta et al., 2018; Kulshreshtha et al., 2019). Investigation of sEV biogenesis pathways has led to reports of various agents that modulate sEV biogenesis/release in recent years (Catalano & O'Driscoll, 2020; Emam et al., 2018; García‐Seisdedos et al., 2020; Khan et al., 2018; Kulshreshtha et al., 2019; Ludwig et al., 2020; Zhang et al., 2020). Previously, one of the main setbacks in identifying potent sEV production modulators was the lack of a sensitive, high throughput system for quantifying sEV production. Generally, sEV quantification requires time‐consuming purification steps involving ultracentrifugation or size exclusion chromatography. These steps eliminate protein contamination that could potentially influence the quantification assay, however, significantly decreases the throughput. The development of a cell‐based assay system utilizing CD63‐GFP allowed for quantitative high‐throughput screening (HTS) of existing drug libraries to identify potent modulators of sEV production (Datta et al., 2018; Im et al., 2019; Zhang et al., 2020). Nonetheless, these assay systems relied on the changes in intracellular CD63‐GFP signals to identify potent activators and inhibitors of sEV production, thus, not directly measuring sEV production.

Therefore, in this study, a rapid, highly sensitive sEV quantification method utilizing Gaussia luciferase (gLuc) reporter protein was developed and used to screen for potential sEV production modulators. gLuc protein fused to sEV marker proteins, CD63 and CD82, were utilised to label the inner spaces of the sEV membrane to quantify sEV production based on the chemiluminescence of the cell supernatant. Because the gLuc fusion proteins could also be present as soluble proteins or as parts of cell debris in the supernatant, pre‐treatment conditions, including centrifugation (to eliminate cells/cell debris) and proteinase K treatment (to eliminate soluble proteins), were evaluated. After confirming the validity of the developed assay, 480 compounds from our in‐house chemical libraries were screened to identify potent regulators of sEV production. The selected hit compounds from the screen were then further validated for their ability to modulate sEV production.

## 2 MATERIALS AND METHODS

### 2.1 Cell culture

Murine melanoma cell line B16BL6 was obtained from RIKEN BioResource Center (Tsukuba, Japan) and was cultured in Dulbecco's modified Eagle's medium (DMEM; Nissui Co., Ltd., Tokyo, Japan) supplemented with 10% heat‐inactivated foetal bovine serum (FBS), 2 g/L D‐glucose and 100 IU/ml penicillin/100 μg/ml streptomycin/2 mM ʟ‐glutamine (PSG; Nacalai Tesque Inc., Kyoto, Japan). Human lung carcinoma cell line A549, human embryonic kidney cell line HEK293 and murine fibroblast cell line NIH3T3 were purchased from the American Type Culture Collection (ATCC; Rockville, MD, USA) and were cultured in DMEM supplemented with 10% FBS and PSG. Murine colorectal cancer cell line Colon26, obtained from the Cancer Chemotherapy Center of the Japanese Foundation for Cancer Research (Tokyo, Japan) and murine macrophage cell line RAW264.7, purchased from ATCC, were cultured in Roswell Park Memorial Institute (RPMI) 1640 medium (Nissui Co., Ltd.) supplemented with 10% FBS and PSG. All cell lines were cultured in 37°C incubator with 5% CO_2_.

### 2.2 Plasmid DNA (pDNA)

Coding sequence of CD63‐gLuc was obtained as previously described (Charoenviriyakul et al., 2018). CD82 mRNA was extracted from RAW264.7 cells and subjected to RT‐PCR to obtain the cDNA sequence. The chimeric sequence of CD82‐gLuc was prepared by using a 2‐step PCR method with the following primers: CD82 Fw: 5′‐ATGCAGATCTTGCAGAATGGGGGCAGGCTGTGTCAAAGTCACCAA‐3′; CD82 Rv: 5′‐GTACTTGGGGACCTTGCTGTAGTCTTCAGAATG‐3′; gLuc Fw: 5′‐AGACTACAGCAAGGTCCCCAAGTACGGTAAGCCCACCGAGAACAACGAAGAC‐3′; and gLuc Rv: 5′‐CTACGCTAGCTTAGTCACCACCGGCCCCCTTGA‐3′. The constructed chimeric sequence was initially subcloned into the BglII/NheI site of the pcDNA3.1 vector (Invitrogen, Carlsbad, CA, USA) and subsequently subcloned into the pROSA‐mcs vector. The promoter and enhancer coding sequences of pBROAD2‐mcs (InvivoGen, San Diego, CA, USA) were amplified by PCR and subcloned into the SdaI/HindIII site of the pCpGfree‐mcs vector (Thermo Fisher Scientific, Waltham, MA, USA) to form the pROSA‐mcs vector. To construct the pROSA constructs encoding the corresponding fusion proteins, the chimeric sequences of CD63‐gLuc and CD82‐gLuc were subcloned into the KpnI/PmeI site of the pROSA‐mcs vector.

Coding sequence of CD63 was subcloned into the pEGFP‐N1 vector (BD Biosciences Clontech, Palo Alto, CA, USA), and the constructed chimeric sequence of CD63‐EGFP was then subcloned into the BamHI/XbaI site of the pcDNA3.1 vector (Invitrogen). The coding sequence of mCherry was prepared from the pPK2‐BAR‐mCherry vector (Takara Bio Inc., Shiga, Japan), and the coding sequence for Lamp2c was obtained as previously described (Matsumoto et al., 2021). The chimeric sequence of mCherry‐Lamp2c was prepared by using a 2‐step PCR method with the following primers: mCherry Fw: 5′‐CTTTCTGTTCCTAGGAGCCGTTCAGTCCAATGCAGGTGTGAGCAAGGGCGAGGAGGA‐3′; mCherry Rv: 5′‐TCTGTCAAATTAACTATCAAACCCTTGTACAGCTCGTCCATGCC‐3′; Lamp2c Fw: 5′‐TTGATAGTTAATTTGACAGA‐3′; and Lamp2c Rv: 5′‐GGGGGGCTTAAGTTACAGAGTCTGATATCCAGCATAGGTC‐3′. To construct the pROSA construct encoding mCherry‐Lamp2c, the chimeric sequence was subcloned into the KpnI/AflII site of the pROSA‐mcs vector.

pDNAs encoding CD63‐gLuc or CD82‐gLuc were individually transfected into B16BL6 cells with polyethyleneimine (PEI) “max” (Polysciences, Warrington, PA, USA). Briefly, 80 μl PEI “max” solution (0.323 mg/ml, pH 8.0) and 10 μg pDNA were individually diluted to 500 μl with 150 mM NaCl, mixed, and incubated at room temperature for 15 min to form the PEI‐DNA complex. This solution was then added to the cells and incubated for 24 h. The medium was changed to Opti‐MEM (Thermo Fisher Scientific) and incubated for an additional 24 h.

### 2.3 Chemistry

KPYC08425 was purchased from ChemDiv (CAS Registry Number: 300402‐36‐2).

KPYC12163 was synthesised in one step from flazin, which was prepared according to literature procedures (Tang et al., 2008) (Figure S1A). The reaction was performed using syringe‐septum cap techniques under argon atmosphere. Wakosil C‐300 was used for flash chromatography. ^1^H NMR spectrum was recorded using a JEOL ECA‐500 spectrometer (JEOL Ltd., Tokyo, Japan) at 500 MHz frequency (Figure S1B). Chemical shifts are reported in δ (ppm) relative to Me_4_Si (in DMSO‐d_6_) as an internal standard. ^13^C NMR spectrum was recorded using a JEOL ECA‐500 spectrometer (JEOL Ltd.) (Figure S1C) and referenced to the residual DMSO signal (in DMSO‐d_6_). Exact mass (HRMS) spectrum was recorded on a JMS‐HX/HX110A mass spectrometer.

Tryptamine (128 mg, 0.799 mmol), EDC·HCl (230 mg, 1.20 mmol), HOBt·H_2_O (184 mg, 1.20 mmol) and triethylamine (0.17 ml, 1.2 mmol) were added to a stirred solution of flazin (123 mg, 0.399 mmol) in DMF (8.0 ml) at 0°C. After stirring for 10 h at room temperature, the mixture was diluted with CHCl_3_. The organic layer was washed with saturated NaHCO_3_ aq. and brine and dried over Na_2_SO_4_. The filtrate was concentrated under reduced pressure to give a residue, which was purified by flash chromatography over silica gel with EtOAc:CHCl_3_:AcOH (80:19:1) to give KPYC12163 as a pale yellow solid (55 mg, 31% yield): mp 252–254°C; ^1^H NMR (500 MHz, DMSO‐d_6_) δ 3.07(t, *J* = 7.4 Hz, 2H), 3.72(td, *J* = 7.4, 5.4 Hz, 2H), 4.72(d, *J* = 6.1 Hz, 2H), 5.49(t, *J* = 6.1 Hz, 1H), 6.64(d, *J* = 2.6 Hz, 1H), 7.00(t, *J* = 7.4 Hz, 1H), 7.09(t, *J* = 7.4 Hz, 1H), 7.29(s, 1H), 7.34(t, *J* = 7.4 Hz, 1H), 7.37(d, *J* = 7.4 Hz, 1H), 7.46(d, *J* = 2.6 Hz, 1H), 7.64(t, *J* = 7.4 Hz, 1H), 7.69(d, *J* = 7.4 Hz, 1H), 7.82(d, *J* = 7.4 Hz, 1H), 8.41(d, *J* = 7.4 Hz, 1H), 8.78(s, 1H), 8.83(t, *J* = 5.4 Hz, 1H), 10.88(s, 1H), 11.47(s, 1H); ^13^C NMR (125 MHz, DMSO‐d_6_) δ 25.3, 55.9, 109.0, 110.9, 111.3, 111.7, 112.2, 112.6, 118.2, 118.4, 120.2, 120.9, 121.0, 121.9, 122.6, 127.2, 128.7, 130.2, 131.31, 131.32, 136.2, 139.5, 141.3, 151.5, 157.2, 164.2, one CH_2_ peak was buried in the solvent signals; HRMS (FAB) m/z: [M + H]^+^ calculated for C_27_H_23_N_4_O_3_, 451.1765; found, 451.1777.

### 2.4 sEV isolation

Conditioned medium of CD63‐gLuc or CD82‐gLuc‐transfected B16BL6 cells were collected and subjected to sequential centrifugation at 300 × *g* for 10 min, 2000 × *g* for 20 min and 10,000 × *g* for 30 min to remove cell debris and large vesicles. Subsequently, the supernatant was passed through 0.2 μm syringe filters and spun at 100,000 *× g* for 1–2 h (Himac CP80WX ultracentrifuge; Hitachi Koki, Tokyo, Japan). The resulting sEV pellets were washed three times with filtered phosphate‐buffered saline (PBS), and the final sEV pellets were resuspended in small volumes of PBS (50–100 μl).

For proteinase K treatment, Proteinase K (ProK; Nacalai Tesque Inc.) was added to the sEV samples to a final concentration of 1 mg/ml and incubated at 37°C for 10 min to digest the surface proteins. Phenylmethylsulfonyl fluoride (PMSF) was then added to a final concentration of 5 mM and incubated at 25°C for 10 min to inhibit ProK activity. The gLuc activity of the sEV samples was measured as described below. The gLuc activity of the ProK‐digested sEV samples was calculated as the percentage of gLuc activity of the untreated sEV samples.

### 2.5 Single‐tube luciferase assay

Untreated and ProK‐treated CD63‐gLuc and CD82‐gLuc labelled sEV samples were lysed with lysis buffer and mixed with sea pansy luciferase assay reagent (PicaGene Dual; Toyo Ink Co., Tokyo, Japan) to measure the chemiluminescence using a luminometer (Lumat LB 9507; EG&G Berthold, Bad Wildbad, Germany).

### 2.6 Chemiluminescence‐based sEV quantification assay

pDNAs were individually transfected into B16BL6 cells with PEI max, as described above, and seeded in a 96‐well plate (2 × 10^4^ cells/well). After 24 h of incubation, the cells were treated with or without 5 mg/ml GW4869 (item no. 13127; Cayman Chemical, Ann Arbor, MI, USA) suspended in Opti‐MEM (Thermo Fisher Scientific) and incubated for an additional 24 h. The conditioned medium of each sample was subjected to sequential centrifugation at 300 × *g* for 10 min, 2000 × *g* for 20 min and 10,000 × *g* for 30 min. At each centrifugation step, ProK‐treated and untreated samples were prepared. Additionally, cell lysates for each sample were prepared by washing the cells once with PBS and subsequently lysing with lysis buffer. The chemiluminescence of all samples was measured via a single‐tube luciferase assay. The gLuc activity of the supernatant was divided by the gLuc activity of the lysate to calculate the lysate‐corrected RLU for each sample. Subsequently, the effect of GW4869 on sEV production was determined by calculating the ratio of the lysate‐corrected gLuc activity of GW4869‐treated samples to the control (DMSO). Additionally, the gLuc activity of the ProK‐digested samples was calculated as the ratio of gLuc activity of the untreated samples.

### 2.7 Construction of CD63‐gLuc stably expressing B16BL6 cell line

pDNA encoding CD63‐gLuc was transfected into B16BL16 cells using Lipofectamine 2000 (Invitrogen). Briefly, 0.5 μg pDNA and 1.5 μl Lipofectamine 2000 were individually diluted in 25 μl Opti‐MEM, mixed, and incubated at room temperature for 20 min to form the Lipofectamine‐DNA complex. This solution was then added to the cells and incubated for 24 h. Cell cloning was initiated by limiting dilution, and the clone with the highest expression of CD63‐gLuc activity was selected. CD63‐gLuc protein expression was confirmed by zymography, as described below, and the validity of the sEV quantification assay was confirmed using GW4869 in the established cell line.

For gLuc zymography, B16BL6 cells stably expressing CD63‐gLuc (B16BL6‐CD63‐gLuc) were lysed using the lysis buffer supplied by the PicaGene Dual Sea Pansy Luminescence Kit (Toyo Ink Co.). Zymography of CD63‐gLuc was performed as previously described (Takahashi et al., 2013). Briefly, cell lysate samples were subjected to 10% sodium dodecyl sulphate‐polyacrylamide gel electrophoresis (SDS‐PAGE) under non‐reducing conditions. The gel was washed twice with 2.5% Triton X‐100 for 30 min, once with PBS for 30 min, and then reacted with the sea pansy luciferase assay reagent (Toyo Ink Co.). Chemiluminescence was detected using the LAS‐3000 imaging system (Fujifilm, Tokyo, Japan).

### 2.8 nanoparticle tracking analysis (NTA)

Culture media of B16BL6 and B16BL6‐CD63‐gLuc subjected to sequential centrifugation at 300 *× g* for 10 min, 2000 × *g* for 20 min, or sEVs isolated from B16BL6‐CD63‐gLuc were used as samples. sEV samples were isolated as described in the previous section, and diluted at 1/20, 1/40, 1/80, 1/160, 1/320, 1/640, 1/1280 ratio using lysis buffer. For each sample, the gLuc activity and the particle numbers were measured via single‐tube luciferase assay and NTA. NTA was performed using ViewSizer 3000 (Horiba Scientific, Kyoto, Japan) with 450 nm 250 mW, 520 nm 12 mW and 635 nm 8 mW lasers; data were analysed using the software provided by the instrument.

### 2.9 WST‐8 cell viability assay

The viability of B16BL6 and B16BL6‐CD63‐gLuc cells were measured using the WST‐8 colorimetric assay (Cell Counting Reagent SF; Nacalai Tesque Inc.) according to the manufacturer's instructions. Briefly, 1 × 10^3^ or 1 × 10^4^ cells of both cell lines were seeded into a 96‐well plate and incubated for 24 h. Necessary reagents were added to the cells and incubated for an additional 2 h to allow for the formation of formazan dye. Absorbance of the samples were read at 450 nm (reference at 620 nm) using the Multiskan FC microplate reader (Thermo Fisher Scientific).

### 2.10 Screening of sEV modulators

Total of 480 compounds selected from our in‐house chemical libraries were used for screening. For each screening, 240 compounds were assayed, and the selected compounds were assayed again to ensure reproducibility of data. B16BL6‐CD63‐gLuc cells were seeded into three 96‐well plate (1 × 10^4^ cells/well) and incubated for 24 h. At the either ends of the 96‐well plates (columns 1 and 12), wells for DMSO and GW4869‐treated cells were prepared to account for variabilities that may arise within or between the 96‐well plates. The cells were treated with 10 μM compound (final DMSO concentration 0.5%) and incubated for an additional 24 h. Conditioned media were collected and centrifuged at 700 × *g* for 1 h at 25°C (Plate Spin; Kubota, Tokyo, Japan), which is equivalent to 2000 × *g* for 20 min, to remove cell debris. Cell lysates were prepared by washing the cells once with PBS and subsequently lysing with lysis buffer. After centrifugation, the supernatants were collected, diluted with lysis buffer and transferred into a 384‐well plate to allow for the simultaneous measurement of the samples. Chemiluminescence was recorded using FDSS/μCELL plate reader (Hamamatsu Photonics K.K., Shizuoka, Japan). Baseline chemiluminescence was recorded initially at 27°C every 5 s; sea pansy luciferase assay reagent (Toyo Ink Co.) was added to the supernatant 2 min after the start of the measurement, and the chemiluminescence was recorded every 5 s for the next 10 min. Recorded gLuc activity data were integrated over the 10 min intervals to determine the integrated RLU of each sample. This integrated RLU of the compound‐treated samples were normalised to the integrated chemiluminescence of the control to determine the RLU_Compound_/RLU_DMSO_ (Figure S2). Compounds with ≥ 2.6‐fold increase or ≤ 0.6‐fold decrease in gLuc activity compared to control were considered to show notable changes in gLuc activity. Twenty‐one compounds in total were selected based on the above criteria; however, one of the compounds was not tested further due to structural similarities with another one of the selected compounds.

Subsequently, supernatant and cell lysate samples of the selected 20 compounds were remeasured via single‐tube luciferase assay using Lumat LB 9507 (EG&G Berthold). Compounds that showed robust and reproducible changes in CD63‐gLuc activity were identified as potential modulators of sEV production.

### 2.11 Evaluation of the effect of the identified compounds on gLuc enzyme reaction

To determine the effect of the candidate compounds on the luciferase enzyme activity, conditioned media of B16BL6 cells treated with 5 or 10 μM of KPYC08425 or KPYC12163, respectively, were collected and mixed with gLuc‐LA labelled sEVs. The gLuc activity of each sample was subsequently measured via single‐tube luciferase assay.

### 2.12 Dose‐response and cytotoxicity assay

For the dose‐response assay, B16BL6‐CD63‐gLuc cells were seeded in a 96‐well plate (5 × 10^4^ cells/ml/well) and incubated for 24 h. The cells were then treated with varying doses (0.3–3 μM) of the candidate compounds and incubated for an additional 24 h. One hundred microlitres of the conditioned media and cell lysate samples were collected as described above for single‐tube luciferase assay.

For the cytotoxicity assay, the remaining conditioned medium was used to measure the release of lactate dehydrogenase (LDH) using the Cytotoxicity LDH Assay Kit‐WST (Dojindo Laboratories, Kumamoto, Japan), according to the manufacturer's instructions. Briefly, the remaining culture medium from each condition was transferred to a 96‐well plate and mixed with the assay buffer to quantify LDH release. The absorbance of the samples was read at 490 nm using a microplate reader (Varioskan Lux; Thermo Fisher Scientific).

### 2.13 sEV protein quantification

B16BL6‐CD63‐gLuc cells were seeded in a 150‐mm dish (4 × 10^6^ cells/dish) and incubated for 24 h. The cells were then treated with 1 or 3 μM KPYC08425 or KPYC12163, respectively. After 24 h incubation, the conditioned medium was subjected to sequential centrifugation, filtration and ultracentrifugation, as described above. Protein concentrations were determined using the Quick Start Bradford protein assay (Bio‐Rad, Hercules, CA, USA).

### 2.14 Transmission electron microscopy (TEM) observations

For observation of sEVs isolated from compound‐treated cells, sEV samples were prepared as described in the previous section. The isolated sEVs were fixed with 4% paraformaldehyde and layered on a carbon/Formvar film‐coated TEM grid (Okenshoji Co., Ltd., Tokyo, Japan) for 20 min at room temperature. After washing with PBS, the samples were treated with 1% glutaraldehyde for 5 min and washed four times with distilled water. Finally, the samples were stained with 1% uranyl acetate for 2 min. Observations were performed using a transmission electron microscope (Hitachi, H‐7650; Tokyo, Japan).

For the observation of ultrathin sections of compound‐treated cells, B16BL6 cells (8.4 × 10^3^ cells/well) were seeded into a Nunc Lab‐Tek Chamber Slide system (Thermo Fisher Scientific) and incubated for 24 h. The cells were treated with 1 μM KPYC12163 or GW4869 and incubated for additional 24 h. The cells were then washed twice with PBS, fixed with a solution containing 4% paraformaldehyde and 2% glutaraldehyde solution overnight at 4°C, and subsequently post‐fixed with 1% osmium tetroxide for 90 min. The cells were then dehydrated with a graded series (50%–100%) of ethanol baths and embedded in epoxy resin. Ultrathin sections were cut with an ultramicrotome and stained with uranyl acetate and lead citrate. Observations were performed using a transmission electron microscope (JEOL, JEM‐1400 Flash; Tokyo, Japan).

### 2.15 Western blotting

Cell lysates were prepared as described above. For western blotting of sEV markers (Alix, Hsp70, CD63 and Calnexin), sEVs and lysate samples (0.5 μg protein/sample) were reduced with 100 mM dithiothreitol (DTT) at 95°C for 3 min and subjected to 10% SDS‐PAGE. The separated proteins were transferred to a polyvinylidene fluoride membrane (PVDF; Merck Millipore, Ltd., Billerica, MA, USA) and blocked with Blocking One reagent (Nacalai Tesque Inc.) for 30 min. The membranes were then incubated for 1 h at 25°C or overnight at 4°C with the following primary antibodies: mouse anti‐AIP1(49/AIP1) antibody (catalogue no. 611620; 1:1000; BD Biosciences, San Jose, CA, USA), rabbit anti‐Hsp70 antibody (catalogue no. 4872s; 1:1000; Cell Signaling Technology, Danvers, MA, USA), anti‐CD63(H‐193) antibody (catalogue no. sc‐15363; 1:200; Santa Cruz Biotechnology, Dallas, TX, USA), and rabbit anti‐Calnexin (H‐70) antibody (catalogue no. sc‐11397; 1:1000; Santa Cruz Biotechnology). The membranes were subsequently incubated for 1 h at 25°C with the following horseradish peroxidase (HRP)‐conjugated secondary antibodies: rabbit anti‐mouse IgG antibody (catalogue no. 61–6520; 1:1000; Thermo Fisher Scientific) and goat anti‐rabbit IgG‐HRP (catalogue no. 7074P2; 1:2000; Cell Signaling Technology). Following incubation, the membranes were washed twice with 0.1% Tween 20 Tris‐buffered saline (TBS‐T), once with TBS, and then reacted with Immobilon Western Chemiluminescent HRP substrate (Merck Millipore Ltd.). Chemiluminescence was detected using the LAS‐3000 imaging system (Fujifilm).

Western blotting of lysosome and autophagosome markers (Lamp2(alias CD107b) and LC3BII, respectively) was performed as described above using the following primary antibodies: mouse anti‐CD107b (Mac‐3, M3/84) antibody (catalogue no. 108501; 1:200; BioLegend, San Diego, CA, USA), rabbit anti‐LC3BII (EPR18709) antibody (catalogue no. ab192890; 1:2000; Abcam, Cambridge, United Kingdom), and rabbit anti‐GAPDH (EPR16891) antibody (catalogue no. ab181602; 1:10,000; Abcam). The following HRP‐conjugated secondary antibodies were utilised for detection: rabbit anti‐mouse IgG antibody (1:1000, Thermo Fisher Scientific) and goat anti‐rabbit IgG HRP (1:2000; Cell Signaling Technology).

### 2.16 Fluorescence microscopy observations

pDNAs encoding CD63‐EGFP and mCherry‐Lamp2c were transfected into B16BL6 cells in a 10:1 ratio with PEI max as described above and seeded in a 6‐well plate (2 × 10^5^ cells/ml/well). After 24 h, the cells were treated with 1 μM KPYC12163 or GW4869 and incubated for additional 24 h. The cells were then washed twice with PBS and fixed with 4% paraformaldehyde solution. The fluorescence signals of CD63‐EGFP and mCherry‐Lamp2c were observed using fluorescence microscopy (Biozero BZ‐X710; Keyence Ltd., Osaka, Japan).

### 2.17 Statistical analysis

Differences among data sets were statistically analysed by Student's *t*‐test for paired comparisons and by Tukey‐Kramer test for multiple comparisons. Values were considered statistically significant at *p*  < 0.05.

## 3 RESULTS

### 3.1 Development and optimisation of a sensitive sEV quantification method using gLuc fusion proteins

**FIGURE 1:**
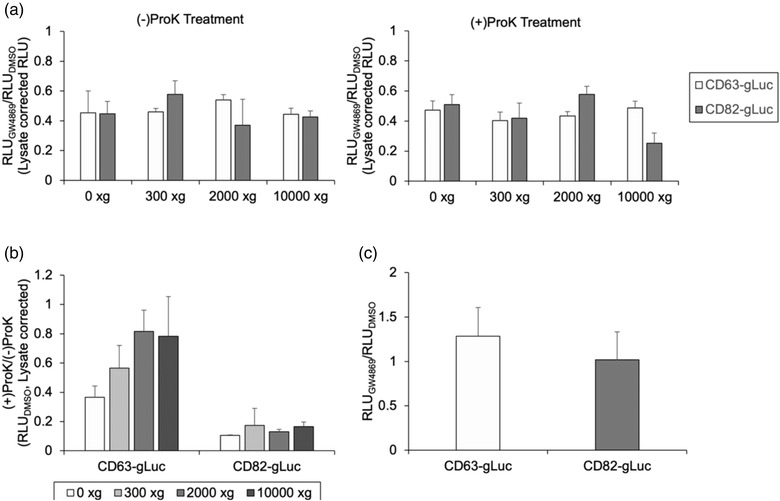
Quantification of sEV production utilizing gLuc fusion proteins. CD63‐gLuc and CD82‐gLuc transfected B16BL6 cells were treated with or without 5 μg/ml GW4869 and the conditioned media were subjected to sequential centrifugation. (a) gLuc activity at each centrifugation step was measured and expressed as the ratio to DMSO (control), left graph represents the gLuc activity of ProK (+) samples, right graph represents the gLuc activity of ProK (−) samples. (b) Ratio of gLuc activities between ProK‐treated and untreated samples when subjected to centrifugation at 2000 × *g* for 20 min. (c) gLuc activity of the cell lysates was measured and expressed as the percentage of control. All data are expressed as the mean ± standard deviation (*n* = 3).

To determine whether the changes in sEV production could be detected by the changes in the supernatant gLuc activity, B16BL6 cells transiently transfected with CD63‐gLuc or CD82‐gLuc constructs were treated with or without GW4869. GW4869 is a neutral sphingomyelinase inhibitor that is known to interfere with sEV secretion and vesicle trafficking (Trajkovic et al., 2008); hence, was utilised as a positive control in the assay. The inhibitory effect of GW4869 on sEV production was confirmed in Figure S3A. The conditioned media were subjected to sequential centrifugation, and the supernatant samples at each centrifugation step were treated with or without ProK to determine the optimal pre‐treatment conditions. Remnants of gLuc fusion proteins maybe present in the supernatant as larger vesicles/cell debris or as a soluble gLuc fusion protein and could potentially impact the estimation of sEV production. Therefore, the gLuc activity of samples after each centrifugation step, and in the presence or absence of ProK was measured to determine the most optimal pre‐treatment condition to accurately estimate the sEV production. The results showed that regardless of the ProK treatment, a reduction in supernatant gLuc activity was observed for all GW4869‐treated groups (Figure 1a). Although both fusion proteins showed a reduction in gLuc activity in the presence of GW4869, comparison of the gLuc activity in samples treated with or without ProK showed a significant reduction in CD82‐gLuc samples treated with ProK (Figure 1b), suggesting a greater presence of soluble proteins in the CD82‐gLuc samples. In contrast, minimal differences in gLuc activity were observed for CD63‐gLuc samples treated with or without ProK when treated with centrifugation >2000 × *g* for 20 min, suggesting minimal presence of soluble proteins after sufficient centrifugation. Based on these findings, CD63‐gLuc was determined to accurately reflect the amount of sEVs in the supernatant samples, even without the additional step of ProK treatment. Further, the gLuc activity of the cell lysate samples was measured to determine whether any changes observed in the supernatant gLuc activity were primarily due to differences in protein expression within the cell (Figure 1c). Cell lysate samples prepared from GW4869‐treated cells had comparable gLuc activity to that of control, suggesting that the reduction in the supernatant gLuc activity resulted from a decrease in sEV production rather than a decrease in the expression of the gLuc fusion proteins.

Because transient transfection may result in variability in the level of fusion protein expression per sample, B16BL6‐CD63‐gLuc cell line was generated for subsequent screening. CD63‐gLuc was selected since it (1) required less pre‐treatment, as indicated by the comparable gLuc activity between untreated and ProK‐treated samples, and (2) exemplified high sensitivities to changes in sEV production caused by the presence or absence of GW4869. Expression of CD63‐gLuc in the stable cell line was confirmed by gLuc zymography, which showed a band near the expected molecular weight of 44 kDa (Figure S4A); the band observed above the 63 kDa mark likely represented the fusion protein between the glycosylated CD63 and gLuc protein. The correlation between the gLuc activity and the particle numbers of the B16BL6‐CD63‐gLuc cell‐derived sEVs was confirmed by luciferase assay and NTA, respectively. Results showed a direct correlation between the gLuc activity and the particle numbers (Figure S4B), which further substantiated the use of gLuc activity data as a convenient estimation for sEV quantification in the gLuc reporter cell line. Stable expression of CD63‐gLuc in B16BL6 cell lines showed no influence on cell viability compared to non‐transfected B16BL6 cells; moreover, no significant differences in the number of particles secreted into the supernatant were observed between B16BL6 and B16BL6‐CD63‐gLuc cell lines (Figure S4C‐D). This suggests that the genetic modification used to establish the stable cell line has minimal impact on the cell and its ability to produce sEVs. Finally, the validity of the assay was confirmed using GW4869 (Figure S4E). A significant reduction in gLuc activity was detected in samples subjected to centrifugation >2000 × *g* for 20 min., which supported the successful development of a sensitive sEV quantification method utilizing CD63‐gLuc fusion protein.

### 3.2 KPYC08425 and KPYC12163 were identified as potential sEV production modulators via screening

**FIGURE 2:**
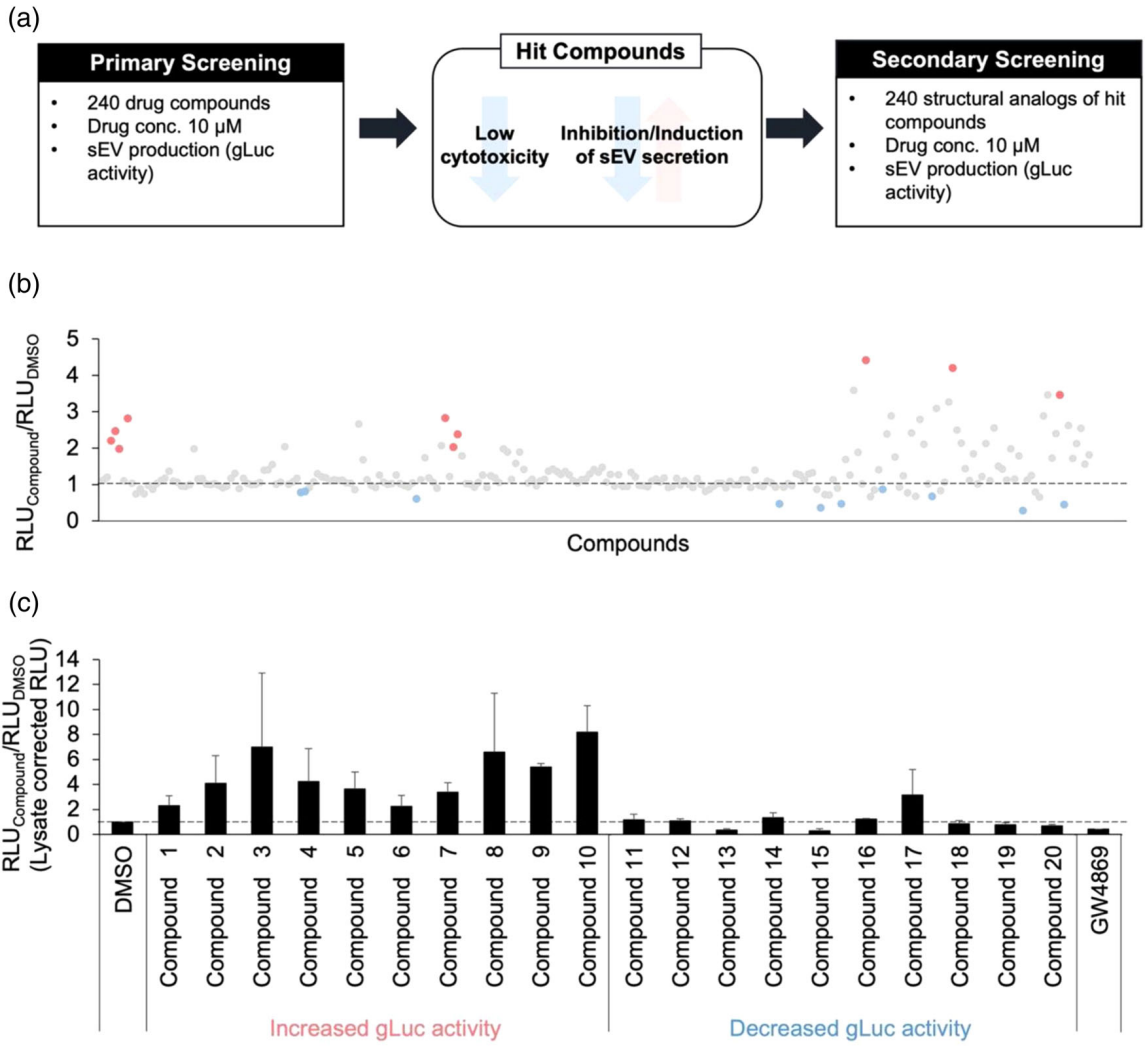
KPYC08425 and KPYC12163 were identified as potential sEV production modulators after two rounds of screening. (a) Schematic flow chart of the screening process. Two‐hundred forty structural analogues of the hit compounds identified from the primary screening were subsequently screened using the developed sEV quantification assay. (b) gLuc activity was measured using FDSS/μCELL. (c) gLuc activity of the selected 20 compounds was remeasured using a luminometer. Results are expressed as the ratio of gLuc activity of the compounds to control, and all data are expressed as the mean ± standard deviation (*n* = 3).

**FIGURE 3:**
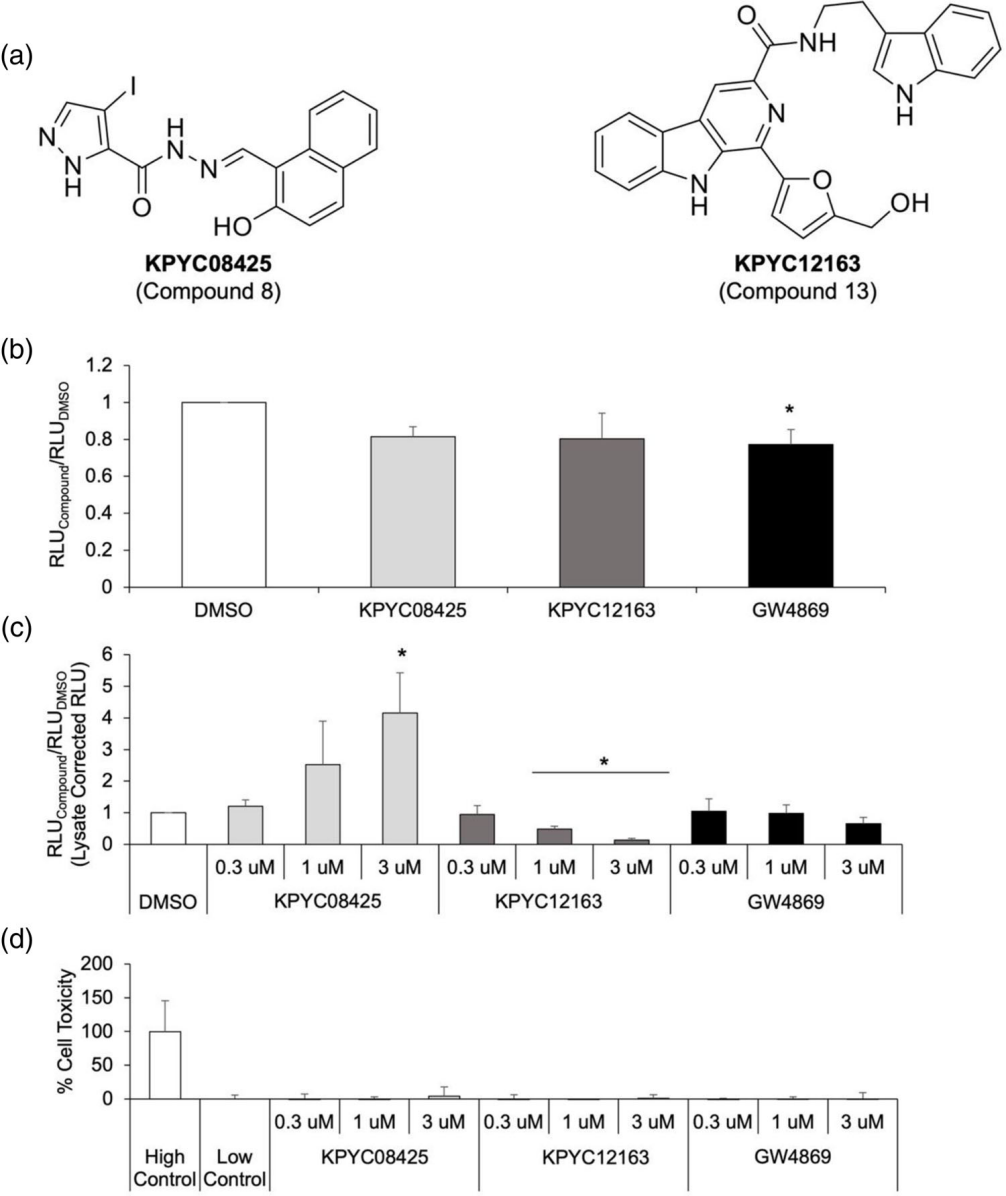
KPYC08425 and KPYC12163 induces dose‐dependent effects on gLuc activity with minor effects on enzyme activity and cytotoxicity. (a) Structural formulae of the identified hit compounds. (b) The effect of compounds on gLuc enzyme activity was determined by mixing the compound‐treated conditioned medium with gLuc‐LA sEVs and measuring the gLuc activity. (c) Dose‐response of the compounds was evaluated by treating the cells with the indicated doses of the compounds and measuring the gLuc activity. The results are expressed as the ratio of the gLuc activity of the compounds to control activity. (d) Cytotoxicity of the compounds at the indicated doses were evaluated by LDH assay. All data are expressed as the mean ± standard deviation (*n* = 3). *p < 0.05 compared to DMSO using Tukey‐Kramer test for (b and c).

Two‐hundred forty compounds selected from our in‐house chemical libraries were initially screened with CD63‐gLuc activity as the readout. This led to identification of four compounds that showed mild effects on sEV production; these four compounds contained either a carbazole, imidazopyridine, thiophen, or phenol scaffolds (Figure S5). To identify more potent sEV modulators, additional 240 structural analogues containing one of the four scaffolds identified were selected from our in‐house chemical libraries and screened (Figure 2a); highlighted in red are compounds that showed increased CD63‐gLuc activity and highlighted in blue are compounds that showed decreased CD63‐gLuc activity (Figure 2b). The gLuc activity of the highlighted compounds was remeasured using single‐tube luciferase assay (Figure 2c) to confirm their stimulatory or inhibitory effect. Based on these assays, cells treated with compounds 3, 8 and 10 showed a notable increase in gLuc activity, while those treated with compounds 13 and 15 showed a substantial decrease in gLuc activity. However, significant reduction in the lysate gLuc activity was observed for compounds 3, 10 and 15, suggesting changes in CD63‐gLuc expression in these compound‐treated cells, rather than a change in sEV production. Thus, compound 8(KPYC08425) and compound 13(KPYC12163) were identified as compounds that can produce potent and reproducible effects on CD63‐gLuc activity (Figure 3a).

To validate the effects of the identified compounds, its effect on the gLuc enzyme reaction, dose‐response and cytotoxicity was assessed subsequently. The addition of the compounds into the reaction mixture hardly changed the chemiluminescence emitted by the gLuc enzyme when reacted with its substrate, coelenterazine (Figure 3b), suggesting that the changes observed in the gLuc activity were not a result of altered enzyme‐substrate interaction. Significant changes in gLuc activity of B16BL6‐CD63‐gLuc cells treated with these two compounds were observed in a dose‐dependent manner, with relatively low cellular toxicity at the investigated doses (Figure 3c and d), supporting its effect on modulating the amount of CD63‐gLuc secreted into the supernatant. Although greater effects were observed at higher doses, increased cellular toxicities became more prominent at doses >5 μM (data not shown); therefore, subsequent experiments were conducted utilizing <3 μM as the dose.

### 3.3 KPYC08425 and KPYC12163 were validated as effective sEV production modulators

**FIGURE 4:**
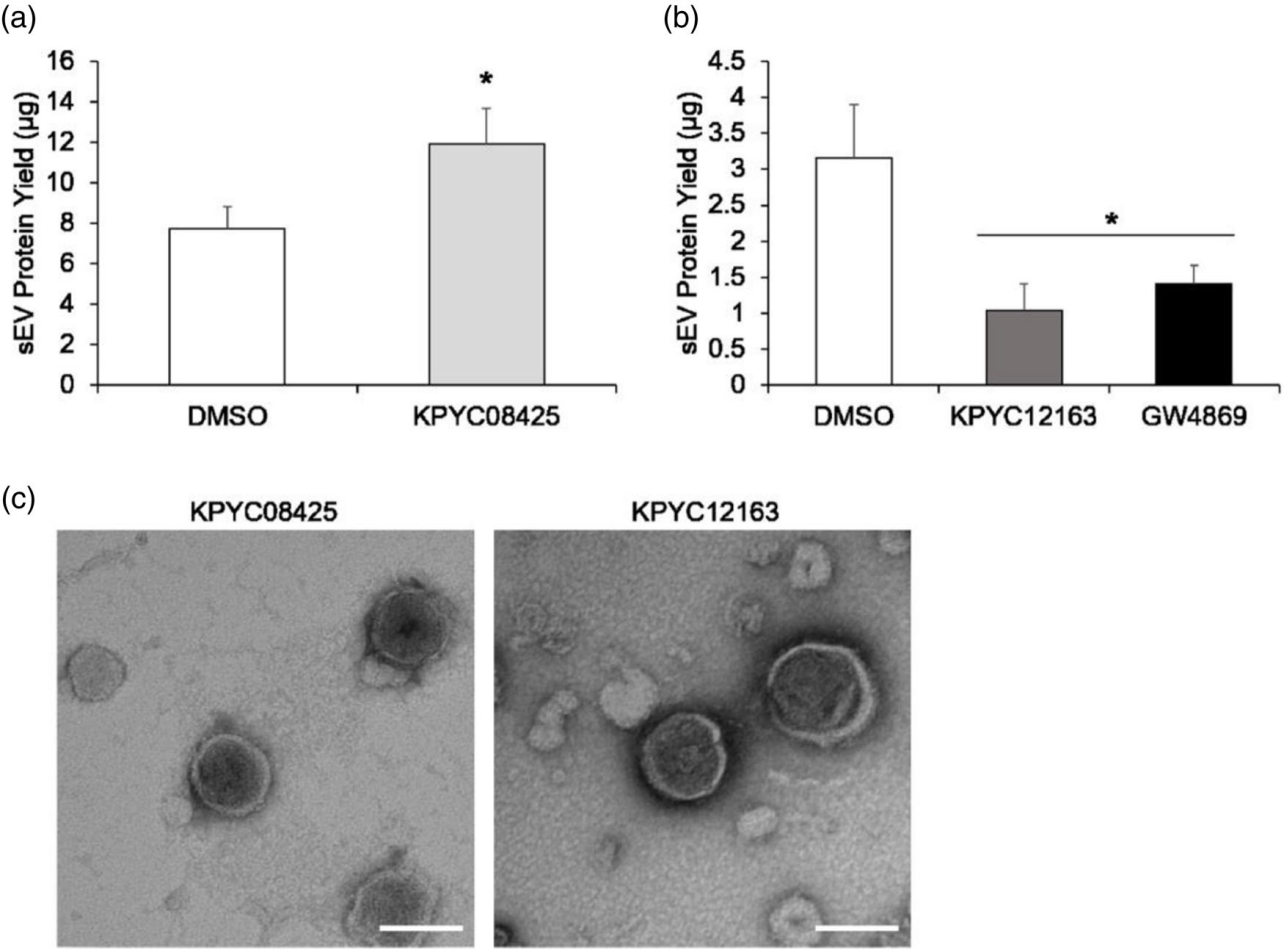
KPYC08425 and KPYC12163 modulated sEV production. sEVs were isolated from KPYC08425 (a) or KPYC12163‐treated cells (b) by ultracentrifugation and quantified by Bradford protein assay. All data are expressed as the mean ± standard deviation (*n* = 3). *p < 0.05 compared to DMSO using Student's *t*‐test for (a) and Tukey‐Kramer test for (b). (c) Isolated sEVs were observed under a transmission electron microscope (TEM) (scale bar = 100 nm).

To confirm the effects of the candidate compounds on sEV production, sEVs were isolated from the conditioned medium of the compound‐treated cells by ultracentrifugation, and the sEV protein yields were assessed. Addition of 1 μM KPYC08425 showed a 1.5‐fold increase in sEV protein yield compared to control (Figure 4a). Addition of 3 μM KPYC12163 resulted in approximately 70% reduction in sEV protein yield compared to control (Figure 4b). Additionally, TEM observation of these isolated sEVs revealed a round‐shaped, bi‐layered vesicle that were approximately 100 nm in size, which are consistent with classic sEV morphology and size (Figure 4c). These findings validated the identified compounds as potent modulator of sEV production. Since KPYC12163 showed a robust inhibitory effect on sEV production, subsequent studies were conducted to investigate its role in sEV production.

### 3.4 KPYC12163 may be inhibiting sEV release thereby stimulating the autolysosomal pathway

**FIGURE 5:**
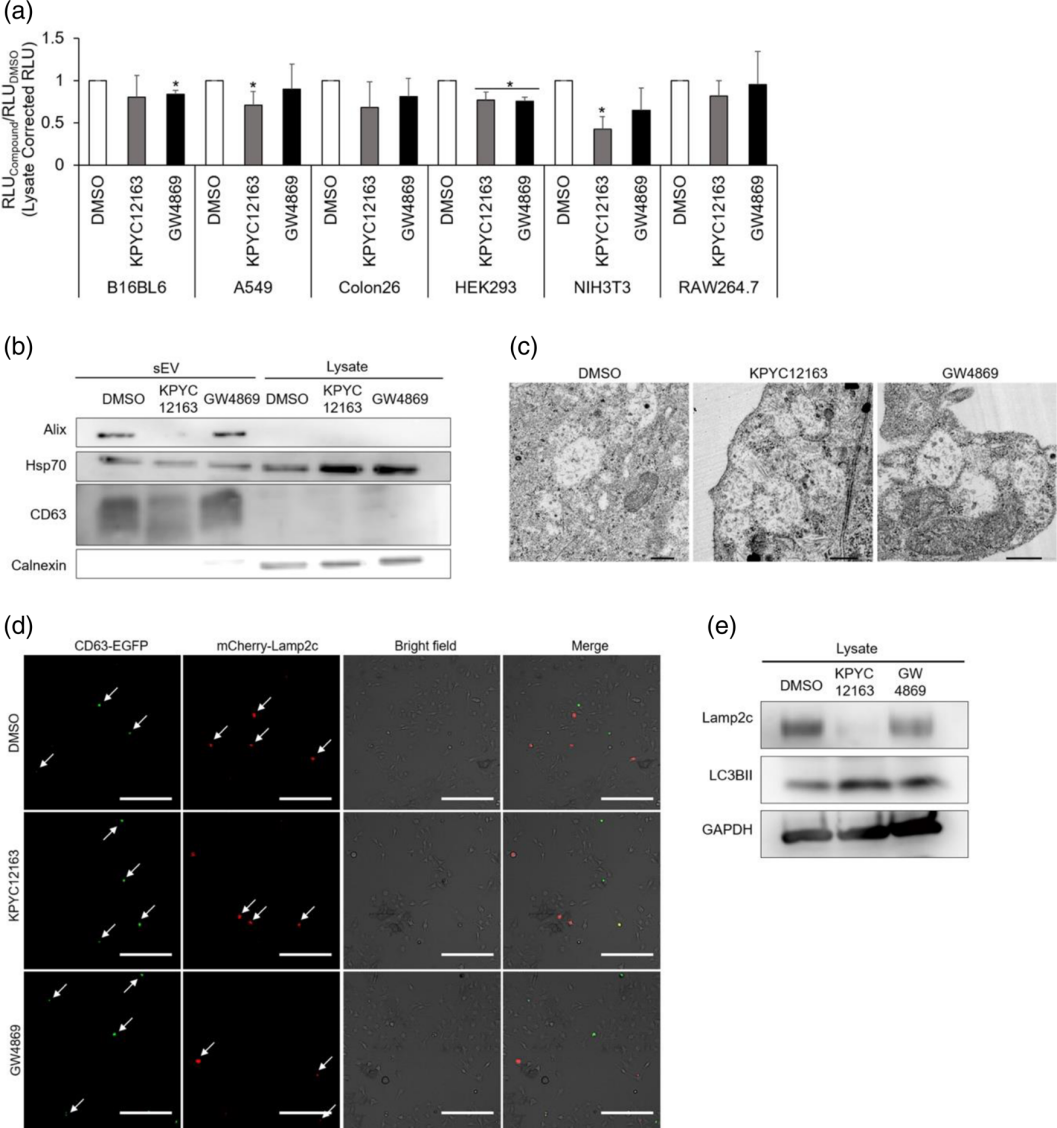
Autolysosomal pathway may be involved in the inhibitory effect of KPYC12163. (a) B16BL6, A549, Colon26, HEK293, NIH3T3 and RAW264.7 cells were treated with 1 μM of KPYC12163 or GW4869 and the gLuc activities of the supernatant and cell lysates were measured. Results are expressed as the ratio of gLuc activity of the compounds to that of the control. All data are expressed as the mean ± standard deviation (*n* = 3). *p < 0.05 compared to DMSO using Tukey‐Kramer test. (b) Western blotting for Alix, Hsp70, CD63 and calnexin in sEVs and cell lysates prepared from control or compound‐treated B16BL6‐CD63‐gLuc cells (0.5 μg protein/lane). (c) Ultrathin sections of B16BL6 cells treated with 1 μM of KPYC12163 or GW4869 as observed by TEM (scale bar = 1 μm). (d) B16BL6 cells transfected with CD63‐EGFP and mCherry‐Lamp2c were treated with 1 μM of KPYC12163 or GW4869 and fixed with 4% paraformaldehyde. The fluorescence signals of CD63‐EGFP and mCherry‐Lamp2c were observed under a fluorescent microscopy (original magnification x200, scale bar = 100 μm). (e) Western blotting for Lamp2c (lysosome marker), LC3BII (autophagosome marker) and GAPDH in control or compound‐treated B16BL6 cell lysates.

First, to determine whether the inhibitory effect of KPYC12163 was cell‐type specific, CD63‐gLuc was transiently transfected into various cell lines, and the changes in gLuc activity upon the addition of the compounds were evaluated. Results showed a decreasing trend in gLuc activity in all cell lines treated with KPYC12163 compared to control (Figure 5a), which suggested that the inhibitory effect of KPYC12163 may occur in multiple different cell lines.

Next, the cargo contents of the isolated sEVs were determined by western blotting. The presence of sEV markers, including Alix, Hsp70, and CD63, and the absence of endoplasmic reticulum marker, Calnexin, was confirmed in the compound‐treated B16BL6‐CD63‐gLuc‐derived sEVs (Figure 5b). sEVs isolated from KPYC12163‐treated cells showed decreased band intensity for all sEV markers compared to control; however, the lysate samples of KPYC12163‐ and GW4869‐treated cells showed increased band intensity for Hsp70. This led to an interesting hypothesis that the compounds were involved in the accumulation of sEVs within the cell, thereby resulting in decreased sEV release.

Generally, sEVs are harboured in multivesicular bodies (MVBs) in the form of intraluminal vesicles (ILVs) within the cell prior to its release. TEM observation of KPYC12163‐treated B16BL6 cells showed increased accumulation of vacuoles within the cell compared to control; accumulation was also observed in GW4869‐treated cells, but to a lesser extent as compared to KPYC12163‐treated cells (Figure 5c). However, due to the similarities in size and morphology, the observed vacuoles could not be distinguished from MVBs by TEM observations. Thus, to evaluate the accumulation of MVBs, CD63‐EGFP and mCherry‐Lamp2c were utilised to label the sEVs, and the changes in fluorescent signals upon the addition of compounds were assessed. Results showed a notable increase in CD63‐EGFP signals, and surprisingly, a decrease in mCherry‐Lamp2c signal upon KPYC12163‐treatment compared to control (Figure 5d). GW4869 showed a comparable signal for CD63‐EGFP and a slight reduction in mCherry‐Lamp2c signal.

**FIGURE 6:**
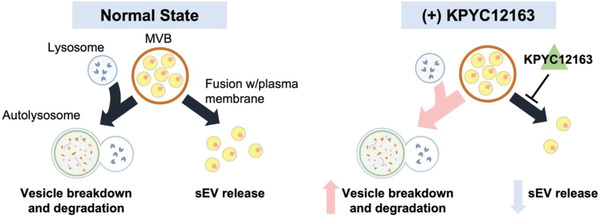
Proposed mechanism of KPYC12163. Under normal state, MVBs either fuse with the plasma membrane to release the sEVs or fuse with the lysosome to form autolysosome for degradation. However, KPYC12163 inhibits the fusion of MVBs with the plasma membrane preventing sEV release. This then results in the accumulation of MVBs, which stimulates the autolysosomal pathway to breakdown the excess MVBs in the cell.

Since Lamp2c proteins also localise in the lysosomal membrane, it was hypothesised that the reduction in mCherry‐Lamp2c signal may be reflective of induced autophagy. Western blot analysis of Lamp2c (lysosome marker) and LC3BII (autophagosome marker) showed decreased band intensity for Lamp2c and increased band intensity for LC3BII in KPYC12163‐treated cell lysates (Figure 5e). Taken together, these results suggested that KPYC12163 inhibited sEV release, thereby causing an accumulation of sEVs within the cell, which then stimulated the induction of autophagy to breakdown the excess sEVs within the cell (Figure 6).

## 4 DISCUSSION

In the current study, a convenient and highly sensitive sEV quantification method was developed by genetically labelling sEVs with gLuc reporter proteins. The use of luciferase fused to sEV marker proteins for sEV quantification has been previously reported (Hikita et al., 2018), and the luminescence intensity was shown to have a strong positive correlation with the number of sEV particles based on NTA (Figure S4). Indeed, the addition of GW4869, an sEV production inhibitor, significantly decreased the gLuc activity in the cell supernatant for both gLuc fusion proteins evaluated (Figure 1), substantiating the gLuc activity as an accurate reflection of the changes in sEV production. However, CD82‐gLuc samples contained more soluble gLuc proteins than CD63‐gLuc, as indicated by the significant reduction in gLuc activity upon ProK treatment (Figure 1). This would necessitate an additional step to remove the excess gLuc proteins for accurate quantification. Therefore, the gLuc fusion protein consisting of CD63, which is a common sEV marker, was selected as the fusion protein for subsequent screening. Nonetheless, sEVs are a heterogeneous group of vesicles that consist of distinct subpopulations depending on their size, cargo contents, and their producing cells, and a subpopulation of sEVs that lack CD63 has been reported previously (Yoshioka et al., 2013). Thus, it is important to recognise that regardless of which fusion protein is selected for sEV labelling, it could result in under‐ or over‐estimation of the sEV amount, and multiple sEV markers should be evaluated for the most precise sEV quantification. Another limitation of this quantification method is that it relies on genetic modification to express the gLuc fusion proteins to label the sEVs for its quantification. Therefore, this method is only applicable to cell lines that can be genetically modified either by transient or stable transfection. This method would not be applicable to certain cells such as primary cells since neither options of genetic modification are feasible for these types of cells.

After two rounds of screening, two hit compounds, namely KPYC08425 and KPYC12163, that regulate sEV production were identified from our in‐house chemical libraries. KPYC08425, which showed a significant increase in gLuc activity, is a hydroxyphenyl hydrazone, which is one of the commonly reported pan‐assay interference compounds (PAINS). PAINS are chemical compounds that interact non‐specifically with target proteins to produce false signals in numerous assays (Baell & Holloway, 2010; Baell & Nissink, 2018). However, evaluation of the effect of KPYC08425 on enzyme activity showed decreased gLuc activity upon addition of the compound (Figure 3), which suggests that the screening results may not be an artefact but an actual reflection of increased sEV production. Purification and quantification of sEVs from cells treated with KPYC08425 showed a 1.5‐fold increase in sEV protein yield (Figure 4), validating KPYC08425 as a potential sEV production inducer. Nonetheless, its effect on sEV production is mild at best, and cytotoxicity becomes an issue at higher doses (data not shown). Hydroxyphenyl hydrazone derivatives have been reported to inhibit cell proliferation through iron sequestration in cancer cells (Li et al., 2017), which may be the cause of increased cellular toxicity. Therefore, further optimisation of the chemical compound is necessary to improve its efficacy.

KPYC12163, which showed a significant reduction in gLuc activity, is not a member of PAINS but still exhibited mild interference with the enzyme activity (Figure 3). However, considering the significant reduction in gLuc activity observed during the screening, it is likely that the compound indeed influenced sEV biogenesis/release. Subsequent dose‐response assay showed not only a dose‐dependent decrease in gLuc activity in the cell supernatant but also an increase in gLuc activity in the cell lysate (data not shown), suggesting that the compound interfered with sEV release. Purification of sEVs showed approximately 70% reduction in sEV protein yield for KPYC12163‐treated cells compared to control (Figure 4), confirming its inhibitory effect on sEV production. All investigated cell lines, including cancerous and non‐cancerous cell lines treated with KPYC12163, exhibited a tendency of decreased gLuc activity (Figure 5), suggesting that the compound likely inhibits a common sEV biogenesis pathway. Additionally, western blot analysis of the sEV markers showed a significant reduction in the band intensity for sEVs isolated from KPYC12163‐treated cells (Figure 5), which implies a decrease in sEV protein yield per μg protein and, potentially, a qualitative change in the sEVs such as altered cargo contents.

Generally, sEVs are derived from the endosomal compartment known as MVBs; the membrane of MVB invaginates to form ILVs, which are secreted into the extracellular milieu upon MVB fusion with the plasma membrane. MVBs are mostly >250 nm in size and are enriched with various proteins such as class II MHC and tetraspanins (Altick et al., 2009; Piper & Katzmann, 2007). TEM observation of compound‐treated cells showed increased accumulation of vacuoles that could be either cytoplasmic vacuolisation of the compounds or MVBs based on their size (Figure 5). Moreover, a slight increase in CD63‐EGFP signals was observed in the compound‐treated cells (Figure 5), which suggests the potential accumulation of MVBs, possibly caused by preventing the MVB‐plasma membrane fusion pathway for sEV release. GW4869 has been reported to decrease the number of fusion events with the plasma membrane by Verweij et al. (2018), which would explain the increased vacuoles observed in the GW4869‐treated cells. Considering that Lamp2c protein is also an sEV‐tropic protein (Théry et al., 2018), it was assumed that like CD63‐EGFP, mCherry‐Lamp2c signals will also increase upon addition of the compound; surprisingly, mCherry‐Lamp2c signals were decreased, and western blot analysis also confirmed the reduction of Lamp2c in KPYC12163‐treated cell lysates.

In addition to its tropism in sEVs, Lamp proteins are also localised on the lysosomal membrane (Eskelinen, 2006). Lysosomes are one of the major players involved in macroautophagy (hereafter referred to as autophagy) that function to degrade cytosolic proteins and damaged organelles by fusion with autophagosomes (Feng et al., 2014). Since sEV biogenesis and autophagy are closely linked by the endo‐lysosomal pathway to maintain cellular homeostasis (Xu et al., 2018), it could be reasoned that the accumulated MVBs (due to inhibited sEV release) induced the autophagy‐lysosome pathway to clear the excess MVBs in the cell. Western blot analysis of LC3BII showed increased band intensity for KPYC12163‐treated cells, suggesting the potential induction of the autolysosomal pathway. Other recently identified sEV inhibitors, such as sulfisoxazole, have also been reported to increase the degradation of MVBs through the autophagy‐lysosome pathway (Im et al., 2019). Nonetheless, further investigation of the changes in the expression of sEV biogenesis/release pathway machineries upon treatment with KPYC12163 is necessary to determine the players involved in the inhibition of sEV release.

## 5 CONCLUSION

By developing a highly sensitive luciferase‐based sEV quantification assay using CD63‐gLuc fusion protein, two novel sEV production modulators, KPYC08425 and KPYC12163, were identified. The developed assay can be easily adapted in various cell lines and requires minimal pretreatment (centrifugation > 2000 × *g* for 20 min), thus, can be utilised as a robust method for high throughput sEV quantification. Compounds identified from the screening showed notable impact on sEV protein yield, with KPYC08425 showing a mild increase and KPYC12163 showing a significant decrease in sEV production. These sEV production regulators can be a useful tool in a wide range of sEV‐based research to understand its biological and pathological functions, as well as potentially, in sEV‐based therapies.

## AUTHOR CONTRIBUTIONS

Aki Yamamoto: Conceptualisation; Data curation; Formal analysis; Methodology; Writing – original draft; Writing – review & editing. Yuki Takahashi: Conceptualisation; Data curation; Formal analysis; Funding acquisition; Methodology; Supervision; Writing – original draft; Writing – review & editing. Shinsuke Inuki: Data curation; Formal analysis; Methodology; Resources; Writing – original draft; Writing – review & editing. Shumpei Nakagawa: Data curation; Methodology. Kodai Nakao: Data curation. Hiroaki Ohno: Supervision; Writing – original draft; Writing – review & editing. Masao Doi: Supervision; Writing – original draft; Writing – review & editing. Yoshinobu Takakura: Supervision; Writing – original draft; Writing – review & editing.

## CONFLICT OF INTEREST

The authors declare no conflict of interest.

## Supporting information

Supplementary information

## References

[jex262-bib-0001] Altick, A. L. , Baryshnikova, L. M. , Vu, T. Q. , & von Bartheld, C. S. (2009). Quantitative analysis of multivesicular bodies (MVBs) in the hypoglossal nerve: Evidence that neurotrophic factors do not use MVBs for retrograde axonal transport. The Journal of Comparative Neurology, 514(6), 641–657. 10.1002/cne.22047 19363811 PMC2861426

[jex262-bib-0002] Baell, J. B. , & Holloway, G. A. (2010). New substructure filters for removal of pan assay interference compounds (PAINS) from screening libraries and for their exclusion in bioassays. Journal of Medicinal Chemistry, 53(7), 2719–2740. 10.1021/jm901137j 20131845

[jex262-bib-0003] Baell, J. B. , & Nissink, J. W. M. (2018). Seven year itch: Pan‐assay interference compounds (PAINS) in 2017—Utility and limitations. ACS Chemical Biology, 13(1), 36–44. 10.1021/acschembio.7b00903 29202222 PMC5778390

[jex262-bib-0004] Becker, A. , Thakur, B. K. , Weiss, J. M. , Kim, H. S. , Peinado, H. , & Lyden, D. (2016). Extracellular vesicles in cancer: Cell‐to‐cell mediators of metastasis. Cancer Cell, 30(6), 27066. 10.1016/j.ccell.2016.10.009 PMC5157696 27960084

[jex262-bib-0005] Catalano, M. , & O'Driscoll, L. (2020). Inhibiting extracellular vesicles formation and release: A review of EV inhibitors. Journal of Extracellular Vesicles, 9(1), 1703244. 10.1080/20013078.2019.1703244 32002167 PMC6968539

[jex262-bib-0006] Charoenviriyakul, C. , Takahashi, Y. , Morishita, M. , Nishikawa, M. , & Takakura, Y. (2018). Role of extracellular vesicle surface proteins in the pharmacokinetics of extracellular vesicles. Molecular Pharmaceutics, 15(3), 1073–1080. 10.1021/acs.molpharmaceut.7b00950 29382201

[jex262-bib-0007] Datta, A. , Kim, H. , McGee, L. , Johnson, A. E. , Talwar, S. , Marugan, J. , Southall, N. , Hu, X. , Lal, M. , Mondal, D. , Ferrer, M. , & Abdel‐Mageed, A. B. (2018). High‐throughput screening identified selective inhibitors of exosome biogenesis and secretion: A drug repurposing strategy for advanced cancer. Scientific Reports, 8(1), 8161. 10.1038/s41598-018-26411-7 29802284 PMC5970137

[jex262-bib-0008] el Andaloussi, S. , Mäger, I. , Breakefield, X. O. , & Wood, M. J. A. (2013). Extracellular vesicles: Biology and emerging therapeutic opportunities. Nature Reviews Drug Discovery, 12(5), 347–357. 10.1038/nrd3978 23584393

[jex262-bib-0009] Emam, S. E. , Ando, H. , Abu Lila, A. S. , Shimizu, T. , Ukawa, M. , Okuhira, K. , Ishima, Y. , Mahdy, M. A. , Ghazy, F. S. , & Ishida, T. (2018). A novel strategy to increase the yield of exosomes (extracellular vesicles) for an expansion of basic research. Biological and Pharmaceutical Bulletin, 41(5), 733–742. 10.1248/bpb.b17-00919 29709910

[jex262-bib-0010] Eskelinen, E.‐L. (2006). Roles of LAMP‐1 and LAMP‐2 in lysosome biogenesis and autophagy. Molecular Aspects of Medicine, 27(5–6), 495–502. 10.1016/j.mam.2006.08.005 16973206

[jex262-bib-0011] Feng, Y. , He, D. , Yao, Z. , & Klionsky, D. J. (2014). The machinery of macroautophagy. Cell Research, 24(1), 24–41. 10.1038/cr.2013.168 24366339 PMC3879710

[jex262-bib-0012] García‐Seisdedos, D. , Babiy, B. , Lerma, M. , Casado, M. E. , Martínez‐Botas, J. , Lasunción, M. A. , Pastor, Ó. , & Busto, R. (2020). Curcumin stimulates exosome/microvesicle release in an in vitro model of intracellular lipid accumulation by increasing ceramide synthesis. Biochimica et Biophysica Acta (BBA) – Molecular and Cell Biology of Lipids, 1865(5), 158638. 10.1016/j.bbalip.2020.158638 31988047

[jex262-bib-0013] Hikita, T. , Miyata, M. , Watanabe, R. , & Oneyama, C. (2018). Sensitive and rapid quantification of exosomes by fusing luciferase to exosome marker proteins. Scientific Reports, 8(1), 14035. 10.1038/s41598-018-32535-7 30232365 PMC6145919

[jex262-bib-0014] Im, E.‐J. , Lee, C.‐H. , Moon, P.‐G. , Rangaswamy, G. G. , Lee, B. , Lee, J. M. , Lee, J.‐C. , Jee, J.‐G. , Bae, J.‐S. , Kwon, T.‐K. , Kang, K.‐W. , Jeong, M.‐S. , Lee, J.‐E. , Jung, H.‐S. , Ro, H.‐J. , Jun, S. , Kang, W. , Seo, S.‐Y. , Cho, Y.‐E. , … Baek, M.‐C. (2019). Sulfisoxazole inhibits the secretion of small extracellular vesicles by targeting the endothelin receptor A. Nature Communications, 10(1), 1387. 10.1038/s41467-019-09387-4 PMC6437193 30918259

[jex262-bib-0015] Khan, F. M. , Saleh, E. , Alawadhi, H. , Harati, R. , Zimmermann, W.‐H. , & El‐Awady, R. (2018). Inhibition of exosome release by ketotifen enhances sensitivity of cancer cells to doxorubicin. Cancer Biology & Therapy, 19(1), 25–33. 10.1080/15384047.2017.1394544 29244610 PMC5790333

[jex262-bib-0016] Kulshreshtha, A. , Singh, S. , Ahmad, M. , Khanna, K. , Ahmad, T. , Agrawal, A. , & Ghosh, B. (2019). Simvastatin mediates inhibition of exosome synthesis, localization and secretion via multicomponent interventions. Scientific Reports, 9(1), 16373. 10.1038/s41598-019-52765-7 31704996 PMC6841733

[jex262-bib-0017] Lässer, C. , Seyed Alikhani, V. , Ekström, K. , Eldh, M. , Torregrosa Paredes, P. , Bossios, A. , Sjöstrand, M. , Gabrielsson, S. , Lötvall, J. , & Valadi, H. (2011). Human saliva, plasma and breast milk exosomes contain RNA: Uptake by macrophages. Journal of Translational Medicine, 9(1), 9. 10.1186/1479-5876-9-9 21235781 PMC3033821

[jex262-bib-0018] Li, F. , Long, L. , Xiao, J. , Wang, C. , Li, W. , Li, S. , Zhao, C. , & Wang, L. (2017). A novel hydroxyphenyl hydrazone derivate YCL0426 inhibits cancer cell proliferation through sequestering iron. Anti‐Cancer Drugs, 28(10), 1131–1140. 10.1097/CAD.0000000000000557 28926421

[jex262-bib-0019] Ludwig, N. , Yerneni, S. S. , Menshikova, E. v. , Gillespie, D. G. , Jackson, E. K. , & Whiteside, T. L. (2020). Simultaneous inhibition of glycolysis and oxidative phosphorylation triggers a multi‐fold increase in secretion of exosomes: Possible role of 2′,3′‐CAMP. Scientific Reports, 10(1), 6948. 10.1038/s41598-020-63658-5 32332778 PMC7181876

[jex262-bib-0020] Matsumoto, A. , Takahashi, Y. , Ogata, K. , Kitamura, S. , Nakagawa, N. , Yamamoto, A. , Ishihama, Y. , & Takakura, Y. (2021). Phosphatidylserine‐deficient small extracellular vesicle (SEV) is a major somatic cell‐derived SEV subpopulation in blood. Iscience, 24, 102839. 10.1016/j.isci.2021.102839 34368655 PMC8326202

[jex262-bib-0021] Piper, R. C. , & Katzmann, D. J. (2007). Biogenesis and function of multivesicular bodies. Annual Review of Cell and Developmental Biology, 23(1), 519–547. 10.1146/annurev.cellbio.23.090506.123319 PMC2911632 17506697

[jex262-bib-0022] Takahashi, Y. , Nishikawa, M. , Shinotsuka, H. , Matsui, Y. , Ohara, S. , Imai, T. , & Takakura, Y. (2013). Visualization and in vivo tracking of the exosomes of murine melanoma B16‐BL6 cells in mice after intravenous injection. Journal of Biotechnology, 165(2), 77–84. 10.1016/j.jbiotec.2013.03.013 23562828

[jex262-bib-0023] Tang, J.‐G. , Wang, Y.‐H. , Wang, R.‐R. , Dong, Z.‐J. , Yang, L.‐M. , Zheng, Y.‐T. , & Liu, J.‐K. (2008). Synthesis of analogues of flazin, in particular, flazinamide, as promising anti‐HIV agents. Chem Biodivers, 5(3), 447–460. 10.1002/cbdv.200890044 18357553

[jex262-bib-0024] Théry, C. , Witwer, K. W. , Aikawa, E. , Alcaraz, M. J. , Anderson, J. D. , Andriantsitohaina, R. , Antoniou, A. , Arab, T. , Archer, F. , Atkin‐Smith, G. K. , Ayre, D. C. , Bach, J.‐M. , Bachurski, D. , Baharvand, H. , Balaj, L. , Baldacchino, S. , Bauer, N. N. , Baxter, A. A. , Bebawy, M. , … Zuba‐Surma, E. K. (2018). Minimal information for studies of extracellular vesicles 2018 (MISEV2018): A position statement of the international society for extracellular vesicles and update of the MISEV2014 guidelines. Journal of Extracellular Vesicles, 7(1), 1535750. 10.1080/20013078.2018.1535750 30637094 PMC6322352

[jex262-bib-0025] Tkach, M. , & Théry, C. (2016). Communication by extracellular vesicles: Where we are and where we need to go. Cell, 164(6), 1226–1232. 10.1016/j.cell.2016.01.043 26967288

[jex262-bib-0026] Trajkovic, K. , Hsu, C. , Chiantia, S. , Rajendran, L. , Wenzel, D. , Wieland, F. , Schwille, P. , Brugger, B. , & Simons, M. (2008). Ceramide triggers budding of exosome vesicles into multivesicular endosomes. Science (1979), 319(5867), 1244–1247. 10.1126/science.1153124 18309083

[jex262-bib-0027] van Niel, G. , D'Angelo, G. , & Raposo, G. (2018). Shedding light on the cell biology of extracellular vesicles. Nature Reviews Molecular Cell Biology, 19(4), 213–228. 10.1038/nrm.2017.125 29339798

[jex262-bib-0028] Verweij, F. J. , Bebelman, M. P. , Jimenez, C. R. , Garcia‐Vallejo, J. J. , Janssen, H. , Neefjes, J. , Knol, J. C. , de Goeij‐de Haas, R. , Piersma, S. R. , Baglio, S. R. , Verhage, M. , Middeldorp, J. M. , Zomer, A. , van Rheenen, J. , Coppolino, M. G. , Hurbain, I. , Raposo, G. , Smit, M. J. , Toonen, R. F. G. , … Pegtel, D. M. (2018). Quantifying exosome secretion from single cells reveals a modulatory role for GPCR signaling. Journal of Cell Biology, 217(3), 1129–1142. 10.1083/jcb.201703206 29339438 PMC5839777

[jex262-bib-0029] Xu, J. , Camfield, R. , & Gorski, S. M. (2018). The interplay between exosomes and autophagy – Partners in crime. Journal of Cell Science, 131(15), jcs215210. 10.1242/jcs.215210 30076239

[jex262-bib-0030] Yáñez‐Mó, M. , Siljander, P. R.‐M. , Andreu, Z. , Bedina Zavec, A. , Borràs, F. E. , Buzas, E. I. , Buzas, K. , Casal, E. , Cappello, F. , Carvalho, J. , Colás, E. , Cordeiro‐da Silva, A. , Fais, S. , Falcon‐Perez, J. M. , Ghobrial, I. M. , Giebel, B. , Gimona, M. , Graner, M. , Gursel, I. , … de Wever, O. (2015). Biological properties of extracellular vesicles and their physiological functions. Journal of Extracellular Vesicles, 4(1), 27066. 10.3402/jev.v4.27066 25979354 PMC4433489

[jex262-bib-0031] Yoshioka, Y. , Konishi, Y. , Kosaka, N. , Katsuda, T. , Kato, T. , & Ochiya, T. (2013). Comparative marker analysis of extracellular vesicles in different human cancer types. Journal of Extracellular Vesicles, 2(1), 20424. 10.3402/jev.v2i0.20424 PMC3760642 24009892

[jex262-bib-0032] Zaborowski, M. P. , Balaj, L. , Breakefield, X. O. , & Lai, C. P. (2015). Extracellular vesicles: Composition, biological relevance, and methods of study. BioScience, 65(8), 783–797. 10.1093/biosci/biv084 26955082 PMC4776721

[jex262-bib-0033] Zhang, H. , Lu, J. , Liu, J. , Zhang, G. , & Lu, A. (2020). Advances in the discovery of exosome inhibitors in cancer. Journal of Enzyme Inhibition and Medicinal Chemistr, 35(1), 1322–1330. 10.1080/14756366.2020.1754814 PMC7717571 32543905

