# Supplementary material for: The identification of novel small extracellular vesicle (sEV) production modulators using luciferase‐based sEV quantification method
Source: J Extracell Biol. 2022 Sep 27;1(9):e62. doi: 10.1002/jex2.62 (PMC11080720; doi:10.1002/jex2.62)

Supplementary Information for:

**The identification of novel small extracellular vesicle (sEV) production modulators using luciferase-based sEV quantification method**

Aki Yamamoto^1^, Yuki Takahashi^*1^, Shinsuke Inuki^2^, Shumpei Nakagawa^3^, Kodai Nakao^1^, Hiroaki Ohno^2^, Masao Doi^3^, Yoshinobu Takakura^1^

**Table of Content**

Legends of Supplementary Figures S1-5

Supplementary Figures S1-5

**Supplementary Figure Legends**

Supplementary Figure S1. Synthesis of KPYC12163. (A) Schematic representation of the synthesis of KPYC12163. (B) ^1^H NMR spectrum for KPYC12163 (500 MHz, DMSO-*d_6_*). (C) ^13^C NMR spectrum for KPYC12163 (125 MHz, DMSO-*d_6_*). EDC·HCl = 1-ethyl-3-(3-dimethylaminopropyl)carbodiimide hydrochloride, HOBt = 1-hydroxybenzotriazole, DMF = *N*,*N*-dimethylformamide.

**Supplementary Figure S2. Schematic of the screening protocol.** 1 × 10^4^ cells/well B16BL6-CD63-gLuc cells were seeded into three 96-well plates and incubated at 37°C incubator with 5% CO_2_ for 24 h. Culture media containing 10 μM of the drug compound (final DMSO concentration 0.5%) was prepared for the 240 compounds to be tested. Seeded cells were washed once with 1xPBS, and the culture media was replaced with 100 μL of drug compound-containing media. The cells were incubated again at 37°C incubator with 5% CO_2_ for 24 h. After incubation the supernatant samples were collected and centrifuged at 700 × *g* for 60 min (equivalent to 2000 × *g* for 20 min); cell lysate samples were prepared by washing the cells once with 1xPBS and subsequently lysing with lysis buffer. After centrifugation, the supernatant samples were diluted by 2-folds using lysis buffer and transferred into a 384-well plate for gLuc reading using FDSS/μCELL plate reader. Afterwards, supernatant and cell lysate samples of compounds that showed notable changes in FDSS/μCELL were re-measured using Lumat LB 9507; cell lysate sample was measured to account for potential changes in gene expression levels. The entire process was performed twice to test for reproducibility of results.

**Supplementary Figure S3. Supplementary data for Figure 1**. (A) Effect of GW4869 on sEV protein yield was determined by treating B16BL6 cells either with or without 5 μg/mL GW4869 and quantifying the isolated sEVs protein amount via Bradford protein assay. (B) Baseline gLuc activity of CD63-gLuc and CD-82 gLuc at each centrifugation steps were measured and expressed as RLU/μL/10s. All data are expressed as the mean ± standard deviation (n=3).

**Supplementary Figure S4.** **Confirmation of the establishment and utility of B16BL6 cell line stably expressing CD63-gLuc for the quantification assay.** (A) Cell lysate of B16BL6-CD63-gLuc was collected, and the expression of CD63-gLuc was confirmed by gLuc zymography. (B) sEVs were isolated from the established stable cell line and the gLuc activity and the particle numbers at each dilution ratios were measured and plotted against each other. The solid line shows the linearity of the fitted curve for gLuc activity vs. sEV particle number. (C) Differing numbers of B16BL6 or B16BL6-CD63-gLuc cells were seeded and the cell viability was measured via WST-8 assay. Data are expressed as the mean ± relative standard deviation (n=3). (D) Conditioned media of B16BL6 and B16BL6-CD63-gLuc were subjected to sequential centrifugation at 300 × *g* for 10 min and 2,000 × *g* for 20 min, and the particle numbers were analyzed via NTA. Data are expressed as the mean ± relative standard deviation (n=3). No s.d. compared to B16BL6 using Student-t test. (E) B16BL6-CD63-gLuc cells were treated with or without 5 μg/mL GW4869 and the gLuc activity at each centrifugation step was measured and expressed as the percentage of control activity. Data are expressed as the mean ± standard deviation (n=3).

**Supplementary Figure S5.** **Structural formulae of the hit compounds from the primary screening.**

**Supplementary Figure S1**


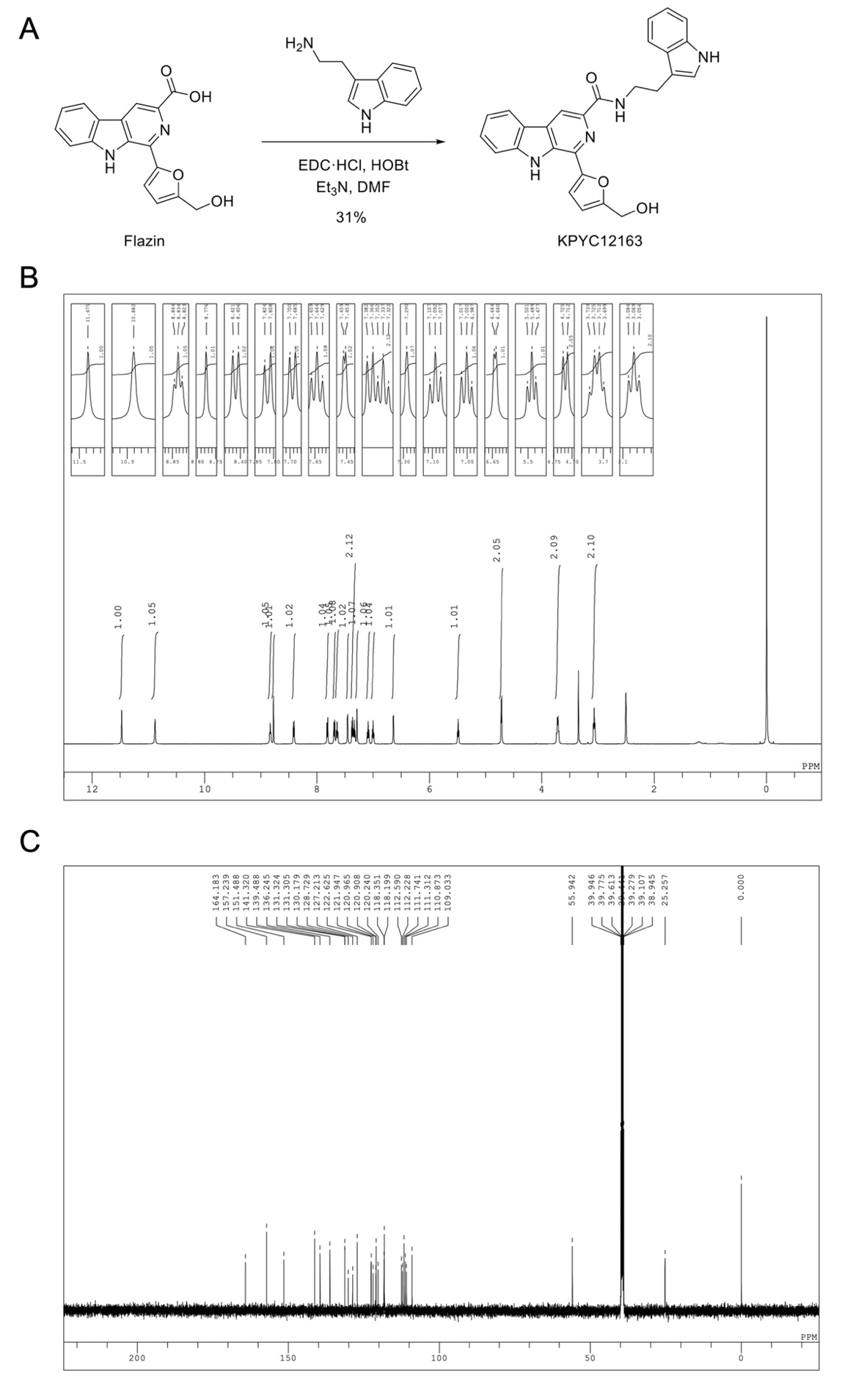


Supplementary Figure S2


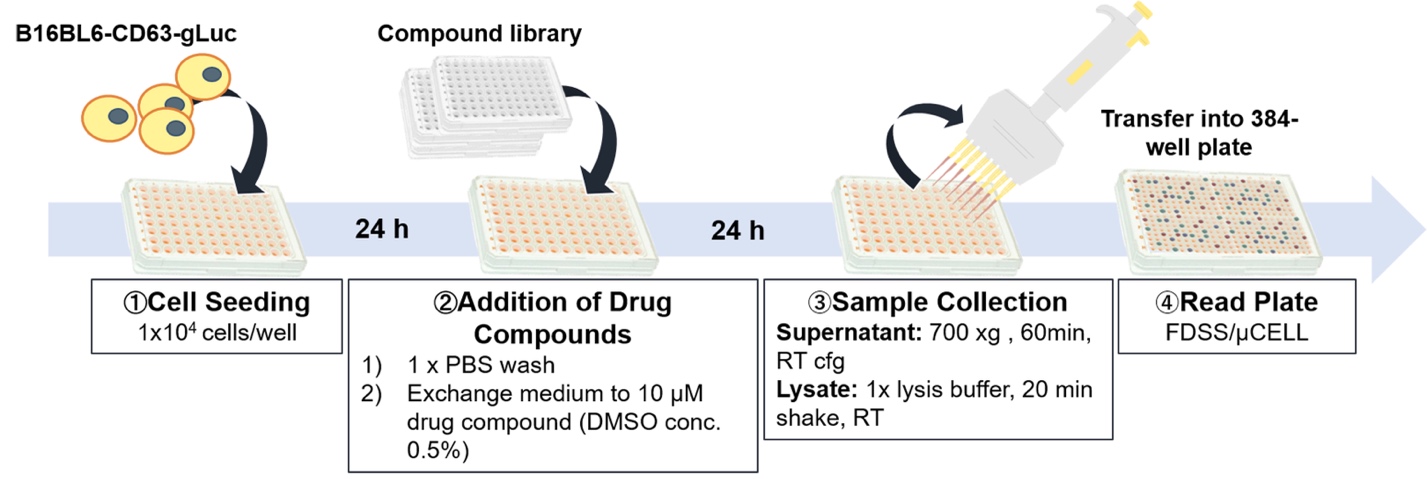


Supplementary Figure S3


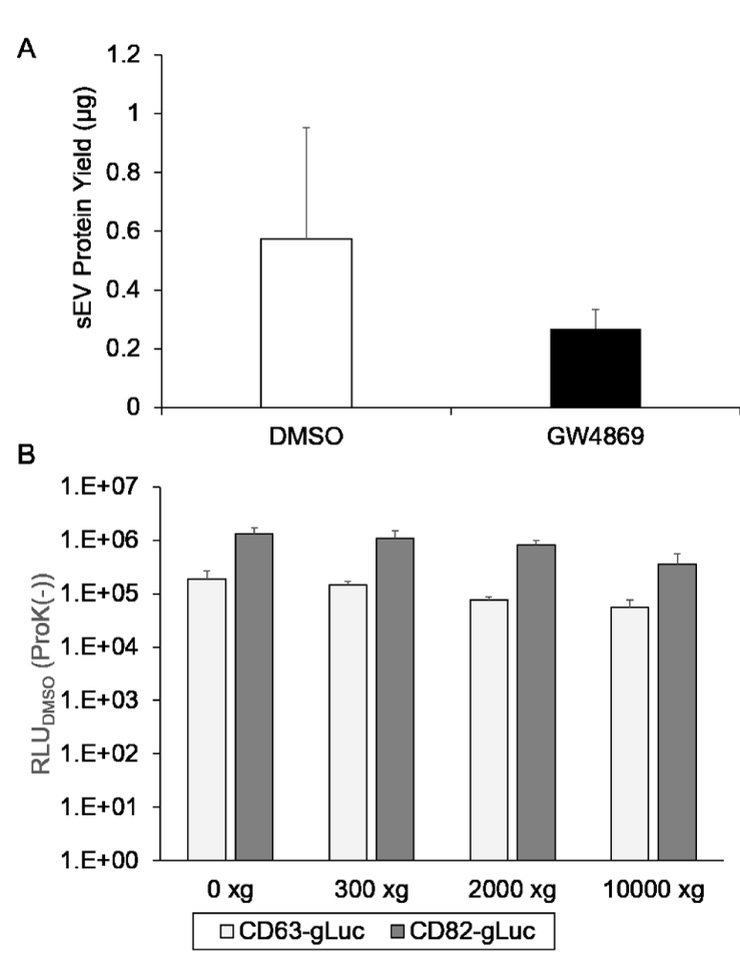


Supplementary Figure S4


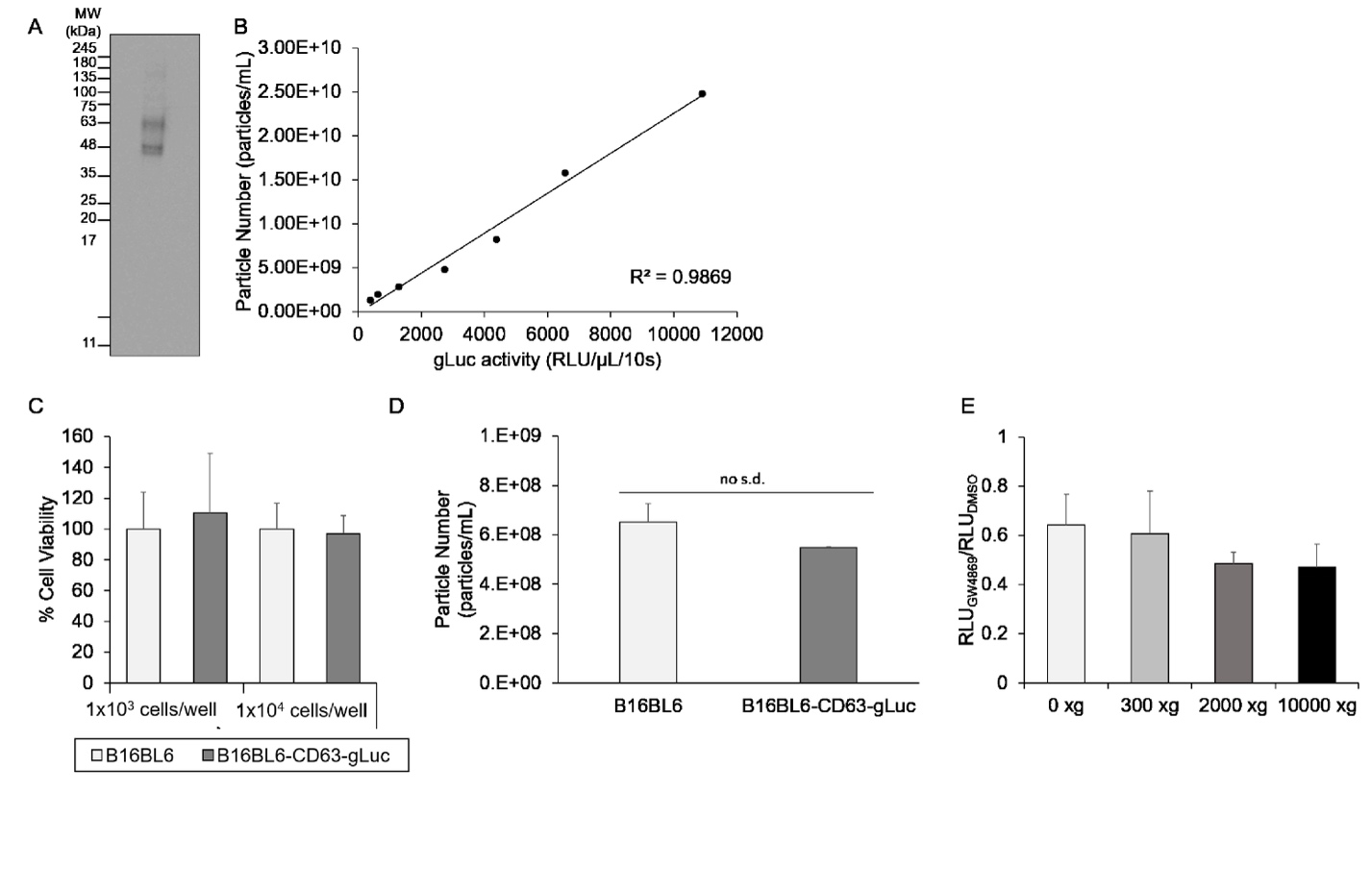


Supplementary Figure S5


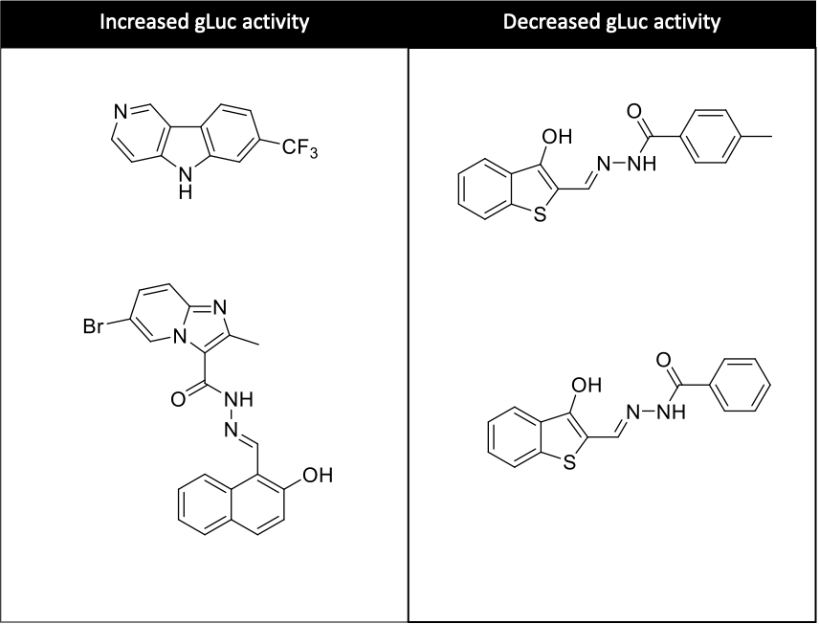

Supplement: Supplementary file 1 — Supplementary information [file JEX2-1-e62-s001.docx]
